# Preparation and Characterization of Conductive/Self-Healing Resin Nanocomposites Based on Tetrafunctional Furan-Functionalized Aniline Trimer Modified Graphene

**DOI:** 10.3390/polym16010090

**Published:** 2023-12-28

**Authors:** Feng Wang, Yichuan Zhang, Su Hu, Xiangyu Zhong, Jiangbo Bai, Yang Zhang, Jianwen Bao

**Affiliations:** 1 AVIC Composite Corporation Ltd., Beijing 101300, China; wangfeng19940925@163.com (F.W.); fc_zhangyic@163.com (Y.Z.); hannah_96@126.com (S.H.); 2 Key Laboratory of Advanced Composite, Composite Technology Center, AVIC Composite Corporation Ltd., Beijing 101300, China; 3 School of Transportation Science and Engineering, Beihang University, Beijing 100191, China

**Keywords:** nanocomposites, graphene, DA reaction, self-healing, electrical conductivity

## Abstract

The nanocomposites with reversible cross-linking covalent bonds were prepared by reacting furfurylamine (FA)-modified diglycidyl ether of bisphenol A (DGEBA) and furfuryl-functionalized aniline trimer-modified graphene (TFAT-G) with bismaleimide (BMI) via the Diels-Alder (DA) reaction. The successful synthesis of the TFAT modifier is confirmed by nuclear magnetic resonance (NMR) hydrogen spectroscopy and IR spectroscopy tests. The structure and properties of TFAT-G epoxy nanocomposites are characterized by scanning electron microscopy (SEM), differential scanning calorimeter (DSC), tensile, and resistivity. The results show that TFAT-G was uniformly dispersed in the resin, and 1 wt% TFAT-G composites increased to 233% for tensile strength, 63% for elongation at break, 66% for modulus, and 7.8 °C for Tg. In addition, the addition of unmodified graphene degrades the mechanical properties of the composite. Overall, the graphene/self-healing resin nanocomposites have both good self-healing function and electrical conductivity by adding 1 wt% modified graphene; this allows for the maintenance of the original 83% strength and 89% electrical conductivity after one cycle of heating repair.

## 1. Introduction

In recent years, polymers with self-healing capabilities have attracted increasing research interest in developing various high-performance materials due to their ability to repair damage and maintain mechanical properties [1,2,3,4,5,6]. The Diels-Alder (DA) reaction, known for its excellent thermal reversibility, is widely used in the design of self-healing materials [7,8,9,10,11]. Due to the reversibility of the DA reaction, self-healing resins can be repaired via the reversible DA reaction after damage. The self-repairing resins maintain the same level of strength after repair, whereas conventional thermosetting resins cannot be repaired after damage. The most commonly used DA reaction system is the furan-maleimide system. The DA bonds break at high temperatures, and the system releases furan and maleimide moieties. Upon cooling to lower temperatures, the furan and maleimide moieties undergo cycloaddition to form covalent bonds, which enables the material to self-heal [11,12].

Incorporation of nanoscale fillers in the resin matrix improves the distinctive properties of composites, such as mechanical, thermal, electromagnetic, and optical behaviors [13,14,15,16,17,18,19]. Nanofillers are mainly affected by the performance of polymer matrix composites in two ways: one is the uniformity of the nanofillers dispersed in the polymer matrix, and another is the interfacial strength between the nanofillers and the polymer matrix. The enhancement of the interfacial strength improves the mechanical and thermo-mechanical properties of the materials. However, the nanofillers are more prone to agglomeration in the polymer matrix, which leads to the deterioration of the material properties. Since the influence of interfacial interactions on the properties of nanocomposites has not yet attracted extensive attention, the enhancement of the mechanical properties is limited with the mere addition of nanofillers. Organic modification of nanofillers not only improves the dispersion in the resin matrix but also enhances the interfacial strength between the two phases. 

Diverse types of modifiers have their respective characteristics, such as the distinctive structures of the modifiers that cause different interfacial interactions between the nanofillers and the resin matrix, and the properties of the nanocomposites would be changed accordingly. The interface of composites assumes the roles of stress transfer and stress dispersion, and its influence on the strength, toughness, and stability of the material should not be neglected. Hesam Amirbeygi et al. introduced aminopropyl trimethoxysilane as an interfacial modifier to the graphene surface, in which they investigated the effect of silane-modified graphene (SGr) with different added mass fractions on the tensile, compressive, and interlaminar shear stress (ILSS) and friction properties of epoxy-based nanocomposites [20]. The results showed that the highest compressive and interlaminar shear properties were obtained with the addition of 0.3 wt% SGr/epoxy nanocomposites, and the coefficients of friction and wear rate were decreased of 40% and 68%, respectively, compared with the pure resin specimens. The tensile, compression, ILSS, and tribological properties of the SGr-epoxy samples with the same mass fraction added were better than those of the unmodified SGr-epoxy samples with the same percentage added. Asad Hameed et al. investigated the effect of different mass fractions of amino- and carboxyl-functionalized multi-walled carbon nanotubes (CNTs) on the mechanical and thermomechanical properties of CNT/epoxy composites [21]. The results showed that the functionalized CNTSs were well dispersed in the epoxy resin without agglomeration. Compared with the pure epoxy resin, the nanocomposites exhibited different degrees of enhanced thermal properties, including higher degradation onset temperatures, higher Tg values, and lower CTE values. Among the different types and added mass fractions of CNTs studied, nanocomposites containing 0.5 wt% amine-functionalized CNTs provided the optimization of thermal and mechanical properties. Amirbek Bekeshev et al. chemically modified the surface of aluminum nitride (AIN) with aminoacetic acid via the preparation of aminofunctionalized AIN and its dispersion in epoxy resin [22]. The results showed that the functionalization of AlN particles by aminoacetic acid ensured better chemical compatibility with epoxy resin components and facilitated the dispersion of AlN particles. The chemical interactions at the epoxy group/AIN interface improved the physico-mechanical properties of the epoxy nanocomposites, and the epoxy composites with the addition of AlN treated with a 5% aminodiacetic acid solution had the best performance. Compared with the epoxy composites with unmodified AlN, the flexural stress and flexural modulus increased by 35% and 80%, respectively, and the tensile strength and tensile elastic modulus increased by 74% and 36%, respectively. The impact strength increased by 133% compared to the pure resin without added AlN.

2D nanomaterials are widely used in self-healing materials to improve mechanical properties and add functional properties [9,23,24,25,26]. Xiao Kuang et al. reacted furan-based multi-wall carbon nanotube furfurylamine (MWCNT-FA) and styrene-butadiene rubber with bifunctional maleimide to generate a covalent bond, which can reversibly crosslink in the rubber composites [27]. The results show that Young’s modulus and toughness of rubber nanocomposites with MWCNT-FA are increased by more than 200–300%. Yuting Zou et al. added 2D MXene nanomaterials to prepare a self-healing composite via furan-based modified bisphenol A epoxy with bismaleimide [28]. The results show that the composites have good reparative properties with 2.80 wt% of MXene nano self-healing layers, which increased the pencil hardness from HB to 5H and the polarization resistance from 4.3 MΩ cm^−2^ to 428.3 MΩ cm^−2^. Graphene is a two-dimensional (2D) material that is a single atom thick (0.335 nm), and the nanosheet layer has a hexagonal lattice structure of sp2 carbon atom arrangement. A large π-electron conjugate structure in the six-membered ring plane gives graphene structural stability and excellent electrical, mechanical, and thermal properties. Due to these specificities, graphene is widely used in nanocomposites [29,30,31].

In the present work, we report a self-healing nanocomposite based on the mechanical properties of modified graphene-reinforced resin with furan functional groups. A tetra-functional furan-containing aniline trimer (TFAT) was synthesized, and graphene (G) was modified to obtain organically modified graphene (TFAT-G). TFAT-G/self-healing conductive nanocomposites were prepared by the DA reaction of furfuramine (FA)-modified DGEBA (DGEBA-FA) and furfuryl-functionalized graphene (TFAT-G) with bismaleimide (BMI). TFAT-G can react with BMI curing agents through furan groups in the modifier and participate in the formation of a resin cross-linking network. TFAT-G plays a triple role as an enhancer, repair agent, and conductive agent in the composite material.

## 2. Materials and Methods

### 2.1. Materials

DGEBA epoxy resin is provided by Nantong Xingchen Synthetic Materials Co., Ltd. (Jiangsu, China). The epoxy value of the DGEBA resin is 0.54. The bismaleimide is sourced from Honghu Bismaleimide New Materials Technology Co., Ltd. (Honghu, China) with a purity of 99%. Epoxy chloropropane, furfuryl alcohol, furfurylamine, p-phenylenediamine sulfate, and aniline are purchased from Shanghai Sigma-Aldrich Chemical Reagents Co., Ltd. (Shanghai, China). Graphene is sourced from Angxing Carbon Materials Changzhou Co., Ltd. (Guangzhou, China). The specific surface area of graphene is 50–200 m^2^/g. All chemicals were used without further purification.

### 2.2. Preparation of Modifiers

**Figure 1:**
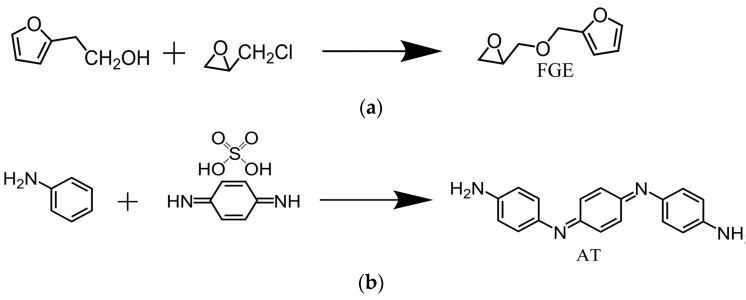
Synthesis mechanism of (**a**) FGE and (**b**) AT.

The synthesis of furfuryl glycidyl ether (FGE) was prepared as follows (Figure 1a): in a 750 mL three-neck round-bottom flask, epichlorohydrin (176.30 g, 2.0 mol) and tetrabutylammonium hydrogen sulfate (6.17 g, 3.5%) was added while maintaining the system temperature in an ice-water bath at 0 °C. Over 30 min, furfuryl alcohol (94.50 g, 0.96 mol) was slowly added dropwise into the above solution. Within 60 min, a 50 wt% NaOH aqueous solution (210 mL) was added to the mixture while controlling the system temperature to not exceed 10 °C. After 3.5 h, the crude product was washed three times with water, the organic layer was collected and dried with anhydrous magnesium sulfate for 24 h. The solvent was removed using a rotary evaporator. The product (FGE) was purified using silica gel column chromatography with ethyl acetate/hexane (5:1) as the mixed solvent. The yield of FGE was 82% [32].

The synthesis of aniline trimers (AT) was carried out as follows (Figure 1b): in a round-bottom flask equipped with a condenser, p-phenylenediamine sulfate (13.30 g), aniline (8.34 g), and 675 mL of 1 M HCl solution were added. The flask was placed in an ice-water bath and mixed well. Ammonium persulfate (18.93 g) was dissolved in 225 mL of 1 M HCl solution. Using a dropping funnel, the ammonium persulfate solution was added dropwise to the above solution at one drop per second. After the addition was completed, it was stirred for 1 h. The reaction mixture was poured into a Buchner funnel and washed thoroughly with deionized water. The product was transferred into a solution of 10 wt% NH_3_·H_2_O and stirred overnight. The filtrate was vacuumed and the product was washed with deionized water. The product was placed in a petri dish, air dried, and then dried overnight at 40 °C under vacuum. The yield was 65% [33].

**Figure 2:**
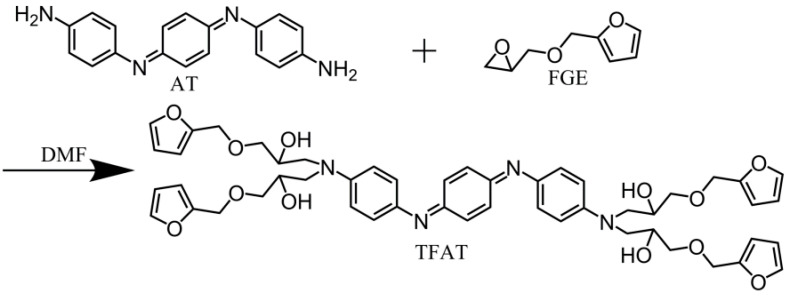
Synthesis mechanism of TFAT.

The synthesis of tetra-functional furan-containing aniline trimer (TFAT) was performed as follows (Figure 2): AT and furyl FGE were dissolved in dry *N*,*N*-dimethylformamide (DMF) with an equimolar ratio of 1:6 (with excess FGE). This results in a 20 wt% DMF solution. The reaction was carried out at approximately 150 °C for 48 h. After the reaction, the solvent was removed with a rotary evaporator. The collected viscous liquid was washed multiple times with toluene, then dried at room temperature under vacuum. The yield of TFAT (viscous liquid) was approximately 80% [19].

### 2.3. Viscosity TFAT-Modified Graphene

**Figure 3:**
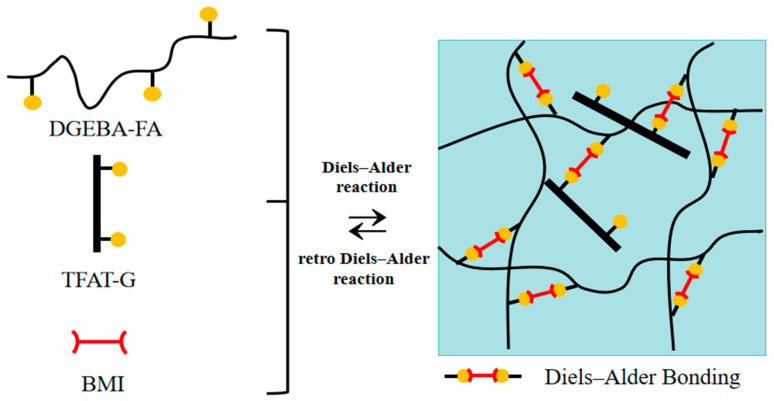
Illustration of utilizing Diels–Alder reaction to synthesize the covalently bonded and reversibly cross-linked nanocomposites.

The preparation process of TFAT-intercalated modified graphene is shown in Figure 3. Into 60 milliliters of a tetrahydrofuran (THF) solvent, 5.31 g TFAT was dissolved. The solution was sonicated for 40 min to ensure complete dissolution of TFAT. Graphene was prepared and added to the TFAT solution in a mass ratio of 3:1 (graphene to TFAT). Sonication of the mixed solution was then performed for 60 min until TFAT intercalated into the graphene layers. The solvent was removed by using a rotary evaporator to evaporate the THF from the solution, leaving behind TFAT-G [34].

### 2.4. The Preparation of Self-Healing Nanocomposite Materials

To prepare the self-healing nanocomposites, 72 g DGEBA and 20.55 g FA were dissolved in 60 g DMF in a sealed conical flask. The solution was heated using an oil bath at 90 °C. The reaction was stirred continuously for a constant 6 h. After ensuring no loss of precipitations, the DMF solution was removed. The polymer/DMF solution (10.17 g, containing 4.425 g polymer) was mixed with 0.789 g BMI. A certain amount of modified graphene (TFAT-G) was dispersed in 1 mL of DMF using ultrasound for 1 h, and then added to the mixture and vigorously stirred while degassed under vacuum. The mixture was poured into a polytetrafluoroethylene mold and cured at 60 °C for 24 h [18].

### 2.5. Characterizations

#### 2.5.1. ^1^H-NMR Analysis

^1^H-NMR spectra were recorded with a 400 MHz AVANCE III NMR spectrometer (Bruker, Karlsruhe, Germany). FGE, AT, and TFAT were dissolved in deuterated CDCl_3_, deuterated DMSO, and deuterated DMF, respectively, for scanning.

#### 2.5.2. FTIR Analysis

The FTIR spectrum was recorded with a NICOLET 6700 spectrometer instrument (Thermo Nicolet, Waltham, MA, USA). The scanning range for infrared spectroscopy is from 400 cm^−1^ to 4000 cm^−1^. 

#### 2.5.3. Thermal Property Analysis 

DSC analysis was performed using a NETZSCH 214 instrument with a flow rate of 20 mL/min (NETZSCH, Selb, Germany). The samples were heated from −40 °C to 80 °C at heating rates of 10 K/min.

#### 2.5.4. The Dispersion State of Graphene Nanocomposites: Characterization 

The dispersion state of graphene nanocomposites was observed by a RENISHAW INVIA confocal micro raman spectrometer (RENISHAW, Gloucestershire, UK). The wave number range of the test is 500 cm^−1^ to 4000 cm^−1^, and the test frequency is 0.1 Hz. 

#### 2.5.5. The Microstructure of Graphene Analysis 

The samples were carried on a 200-mesh copper net, and the TEM images were obtained by JEM-1011 transmission electron microscopy (JEOL, Tokyo, Japan).

#### 2.5.6. Graphene Nanocomposites Repairability Characterization

The surfaces of the samples were examined via a FEI Quanta FEG 250 scanning electron microscope (FEI, Hillsboro, OR, USA). The surfaces were sputter-coated with gold before taking the scanning electron micrographs. 

#### 2.5.7. Mechanical Properties

The mechanical properties were tested on an Instron 5967 instrument (INSTRON, Boston, MA, USA). The test standards for tensile were GB/T 1040 [35], with a crosshead speed of 2 mm/min. Dumbbell sample size is 20 mm × 4 mm × 2 mm (the exact size of each spline is measured before testing). At least five parallel samples were measured for each component and averaged. 

#### 2.5.8. Electrical Properties

The BEST-121 resistivity tester was used to test the resistivity of nanocomposites (Beiguang Instrument and Equipment Company, Beijing, China).

## 3. Results

### 3.1. Characterization of TFAT Modifiers

**Figure 4:**
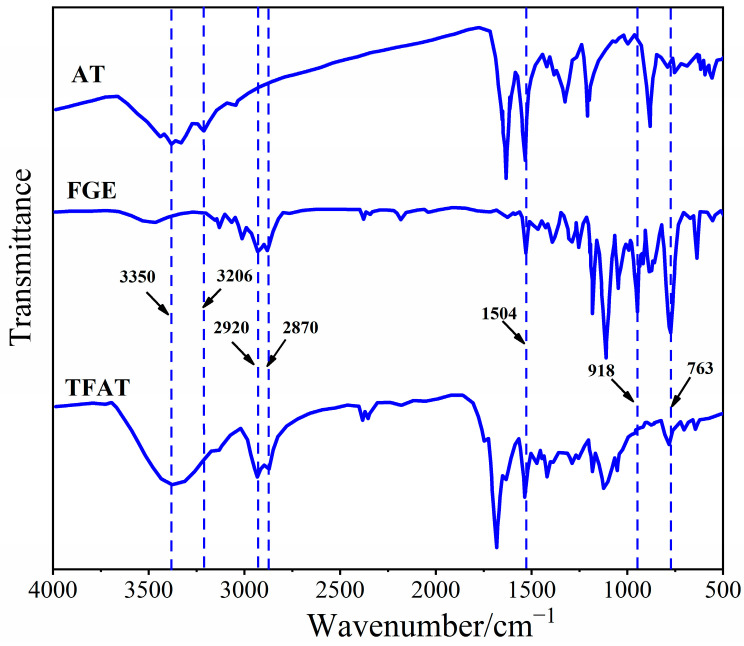
Infrared spectra of AT, FGE, and TFAF.

The synthesized AT, FGE, and TFAT are analyzed by FTIR (Figure 4). The absorption peaks of FGE and TFAF at 763 cm^−1^ are characteristic of the furan ring. The peaks of AT and TFAT at 1504 cm^−1^ are characterized as the benzene ring. The peaks at 2870 cm^−1^ and 2920 cm^−1^ are the stretching vibrationa of methylene. The stretching vibrationa of the methylene groups are seen at 2870 cm^−1^ and 2920 cm^−1^. The absorption peaks at 3206 cm^−1^ and 918 cm^−1^ are the amino group of AT and the epoxy group of FGE, respectively. Due to the addition reaction between the amino and epoxy groups, the absorption peaks of the amino and epoxy groups in TFAF disappeared, and the generated –OH group by the formation reaction appeared at 3350 cm^−1^.

**Figure 5:**
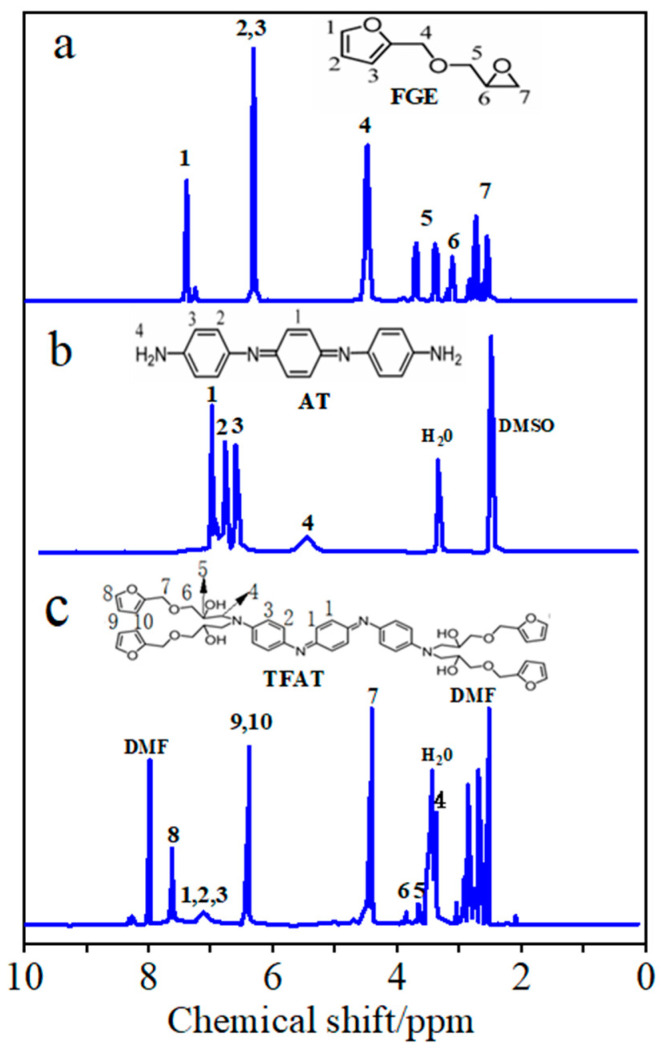
^1^H NMR spectra of (**a**) FGE, (**b**) AT, and (**c**) TFAT.

From the NMR hydrogen spectrum of FGE (Figure 5a), the signal peaks with chemical shifts at 2.65 ppm and 2.75 ppm are characteristic peaks of protons (7) in the ethylene oxide ring, while the signal peaks with chemical shifts at 3.42 ppm and 3.85 ppm are characteristic peaks of –CH_2_ (5) in the ethylene oxide ring. The signal peak with chemical shift at 3.22 ppm is the characteristic peak of the proton of –CH adjacent to the ethylene oxide ring (6). The chemical shifts at 6.2, 6.3, and 7.4 ppm correspond to protons 2, 3, and 1 in the furan ring, respectively. In addition, the signal at 4.5 ppm corresponds to the proton of –OCH_2_ that is partially attached to the furan ring by the glycidyl ether (4). Under the solvent condition of CDCl_3_, the signal at 7.26 ppm is the solvent peak.

In the NMR hydrogen spectrum of AT (Figure 5b), the signal peak at 5.50 ppm is attributed to the aniline trimer-terminal amine proton hydrogen (4). The three signal peaks in the 6.5–7.2 ppm interval are the characteristic peaks of the protons on the benzene ring of the aniline trimer as well as on the quinone ring (1,2,3), and the solvent peak of DMSO is at 2.5 ppm.

As shown in the TFAT NMR hydrogen spectrum (Figure 5c), the chemical shift at 6.88 −7.31 ppm exhibits three signal peaks as aniline trimer benzene ring characteristic peaks (1, 2, 3). Chemical shifts at 6.42 ppm and 7.61 ppm were characteristic peaks for furan group protons (8, 9, 10). The signal peak at 4.50 ppm of chemical shift is the proton characteristic peak of –CH_2_O– attached to the furan ring (7). The characteristic peak at 3.42 mm chemical shift is the proton characteristic peak of –NCH_2_– (4), which has some overlap with the signal peak of water. The characteristic peak at 3.78 ppm chemical shift is the proton peak of –OCH_2_CH(OH) (6), and the characteristic peak at 3.62 ppm chemical shift is the proton peak of –OCH_2_CH(OH) (5).

### 3.2. Characterization of TFAT-Modified Graphene

**Figure 6:**
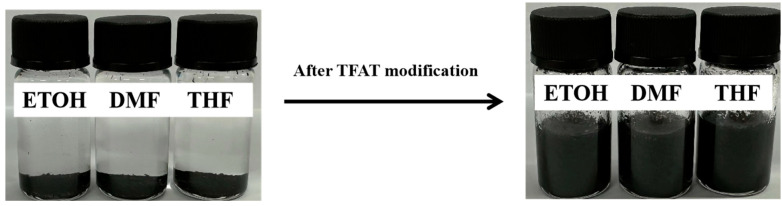
Dispersion state of graphene after TFAT modification in dissolution.

Unmodified graphene flakes have la ow organic degree and poor compatibility with organic matter. Due to the van der Waals force between graphene sheets, unmodified graphene sheets are easily agglomerated and difficult to disperse in organic solvents. The modified graphene with organic matter has higher compatibility and better dispersion in organic matter. Utilizing aromatic organic compounds can effectively modify graphene with π-π interaction. It not only improves the dispersion of graphene but also gives graphene aromatic stability and hydrophobic properties, which expand the application range of graphene. In this study, graphene is modified and dispersed by TFAF, and the graphene is uniformly dispersed through strong π-π conjugation between graphene and TFAF. As shown in Figure 6, the unmodified graphene precipitated after being placed in three organic solvents: ethanol (ETOH), *N*,*N*-dimethylformamide (DMF), and tetrahydrofuran (THF) for 24 h. After adding aniline trimers as dispersants, the graphene still dispersed evenly after being placed in three organic solvents for 24 h without sedimentation or agglomeration.

**Figure 7:**
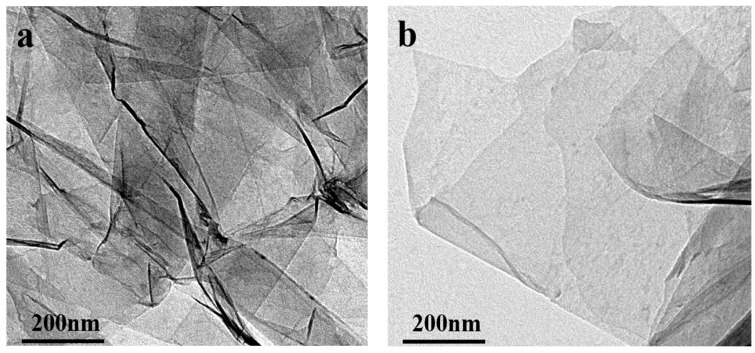
TEM photos of graphene before and after modification (**a**) unmodified graphene, (**b**) TFAT-modified graphene.

From the TEM photos of graphene before and after modification, the unmodified graphene has more lamellae stacked together (Figure 7a). After modification, TFAF was inserted into the interlayers of graphene, and the stacked lamellae were exfoliated by ultrasonic treatment, and the lamellar stacking phenomenon was significantly improved (Figure 7b). Meanwhile, the TFAF-modified graphene showed better compatibility with resin than unmodified graphene. From the changes in the dispersion of graphene before and after modification in solvent and TEM photos, TFAT is an effective organic modifier for graphene.

### 3.3. Dispersion State of Graphene in Resin before and after Modification

**Figure 8:**
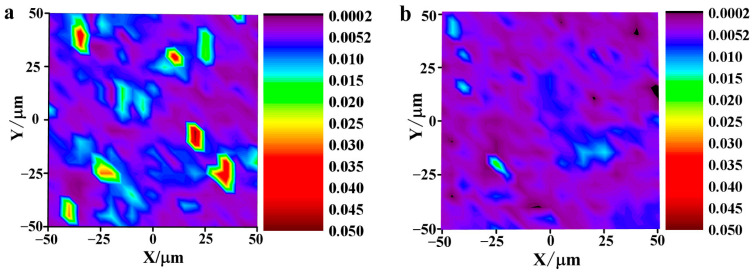
Graphene/epoxy Raman photo (**a**) unmodified graphene, (**b**) TFAF-modified graphene.

The Raman photographs of TFAT-modified and unmodified graphene/resin nanocomposites (Figure 8). The overall dispersion of graphene in the resin matrix before modification is poor, and the phenomenon of graphene lamellae accumulation occurs. The dispersion is improved after TFAF-modified graphene in the epoxy resin matrix, and the overall distribution is more uniform without graphene lamellae or the agglomeration phenomenon.

### 3.4. Characterization of Thermal Property of TFAT-G/Resin Nanocomposites

**Figure 9:**
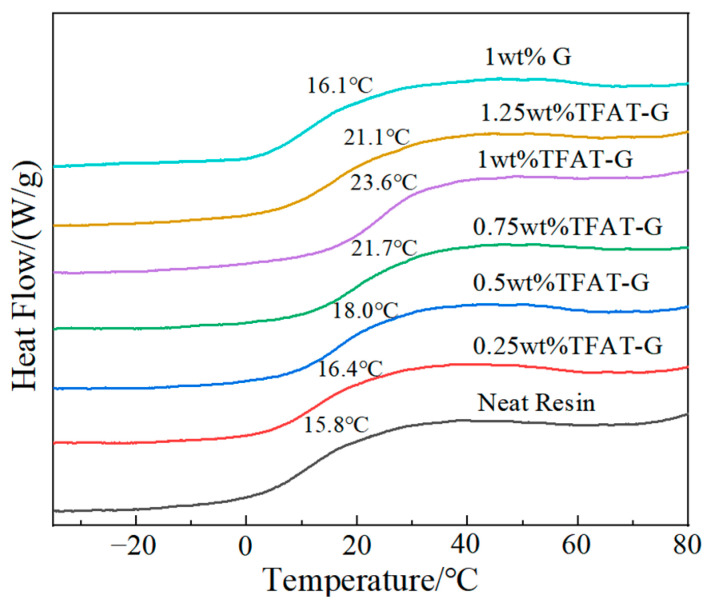
DSC curve of resin nanocomposites.

The epoxy resin graphene nanocomposites are tested by DSC, and the Tg of the pure resin is 15.8 °C (Figure 9). The addition of TFAF-G substantially increased the Tg of the epoxy resin, which reaches a maximum of 23.6 °C when the mass fraction is 1 wt%. TFAF-G contains functional groups that can participate in the curing reaction, which makes the graphene lamellae linked to the epoxy resin matrix through chemical bonding. Also, the binding effect on the resin matrix is noticeably intensified, which causes a significant increase in the Tg of the epoxy resin. When the TFAT-G content was increased to 1.25 wt%, the Tg of the composites was 21.1 °C, which was decreased compared with 1 wt% TFAT-G composites. The excess TFAT-G consumed too much BMI curing agent to reduce the cross-linking degree of the resin matrix, which ultimately led to a decrease in the Tg of the composites. The Tg of the nanocomposite with 1 wt% unmodified graphene is 16.1 °C, which is almost consistent with the pure resin. The unmodified graphene is ineffective at the glass transition temperature of the nanocomposite.

### 3.5. Characterization of Tensile Properties of TFAT-G/Resin Nanocomposites

**Figure 10:**
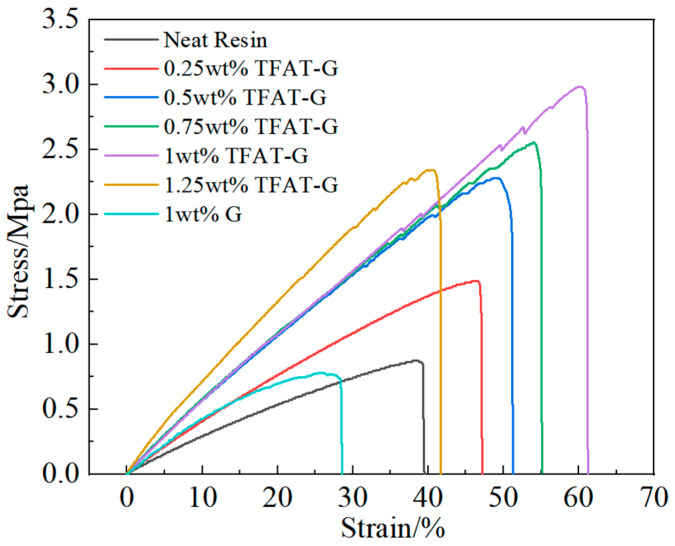
Stress-strain curve of resin nanocomposites.

The pure epoxy resin composites and TFAT-G/resin nanocomposites are analyzed in stress-strain (Figure 10 and Table 1). The tensile strength of the nanocomposites continuously increased with the higher content of TFAT-G. The maximum enhancement in tensile strength was up to 233% as 1 wt% TFAT-G was added to composites. The addition of TFAT-G not only improved the tensile strength of the epoxy resin but also resulted in a significant increase in elongation at break and modulus. The 1 wt% TFAT-G composites resulted in a maximum elongation at break of 63%, attributed to the functional groups on the TFAT molecules. They participate in the curing reaction and construct chemical bonds between the graphene lamellae and the epoxy resin matrix. The stress could be effectively conducted as a result of strong interfacial forces. Compared with the pure resin, the tensile strength of the nanocomposite with 1 wt% unmodified graphene decreased by 12%, and the elongation at break decreased by 26%. As a result of the poor dispersion of unmodified graphene in the resin matrix and the agglomeration phenomenon, there is a deterioration effect on the material properties. When the content of TFAT-G is 1.25 wt%, the modulus of the composite is the highest, which is 171% of the pure resin modulus. It is typical to add graphene to epoxy resin for improvement of modulus since the construction of strong interfacial interaction, in which the reinforcing effect of graphene is efficiently reflected. At the same time, due to the failure of an effective interfacial connection between the unmodified graphene and the resin matrix, the tensile modulus was only 83% with the addition of 1 wt% TFAT-G. 

### 3.6. Characterization of Conductive Properties of TFAT-G/Resin Nanocomposites

**Figure 11:**
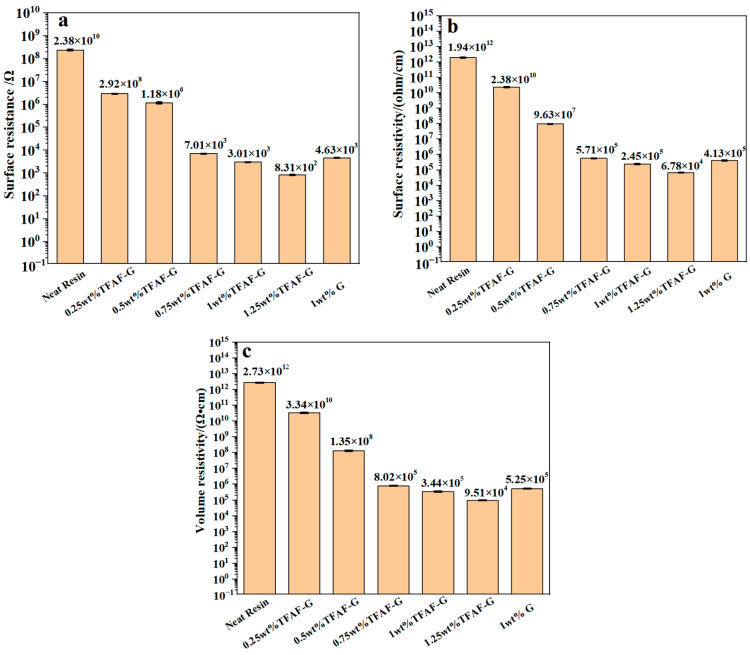
Conductive properties of resin nanocomposites with distinctive wt% of TFAF-G modified (**a**) surface resistance, (**b**) surface resistivity, and (**c**) volume resistivity.

The electrical conductivity data of TFAT-G/resin nanocomposites is shown in Figure 11. The surface resistance, surface resistivity, and volume resistivity of the pure epoxy resin were 2.38 × 10^10^ Ω, 1.94 × 10^12^ (Ω/cm^2^), and 2.73 × 10^12^ (Ω·cm), respectively. As the TFAT-G content increased, the electrical conductivity of the epoxy resin presented substantial enhancement. When 1.25 wt% was added, the surface resistance, surface resistivity, and volume resistivity decreased to 8.31 × 10^2^ Ω, 6.78 × 10^4^ (Ω/cm^2^), and 9.51 × 10^4^ (Ω·cm), respectively. The homogeneous dispersion of graphene in the resin causes a reduction in the conductive threshold value. Only a small amount of graphene improved the conductive properties of the material. Due to the poor dispersion effect of unmodified graphene in the resin matrix, the conduction effect of unmodified graphene nanocomposites was pooer than that of nanocomposites with the same quality of TFAT-G.

### 3.7. Characterization of the Reparative Properties of TFAT-G/Resin Nanocomposites

**Figure 12:**
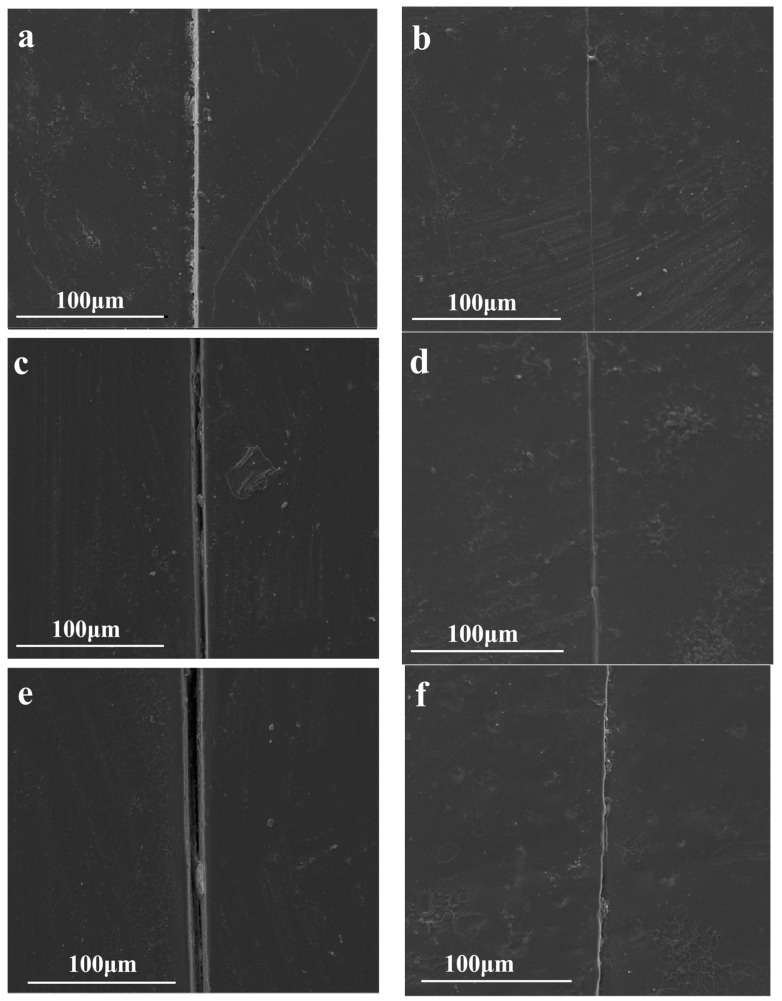
SEM pictures of TFAT-G/Resin nanocomposite (**a**) neat resin, (**b**) neat resin after 1 repair cycle, (**c**) resin with 1 wt% TFAT-G, (**d**) resin with 1 wt% TFAT-G after 1 repair cycle, (**e**) resin with 1 wt% G, (**f**) resin with 1 wt% G after 1 repair cycle.

All the samples were fully cut and repaired by heating after being fractured. TFAT-G/resin nanocomposite section repair was observed by SEM (Figure 12). Because of the furan-bismaleimide Diels-Alder covalent bond in the resin, it has a certain ability to self-heal by heating. The neat resin, the nanocomposites with 1 wt% G, and the nanocomposites with 1 wt% TFAT-G were re-healed after being heated to 150 °C for 30 min. 

**Figure 13:**
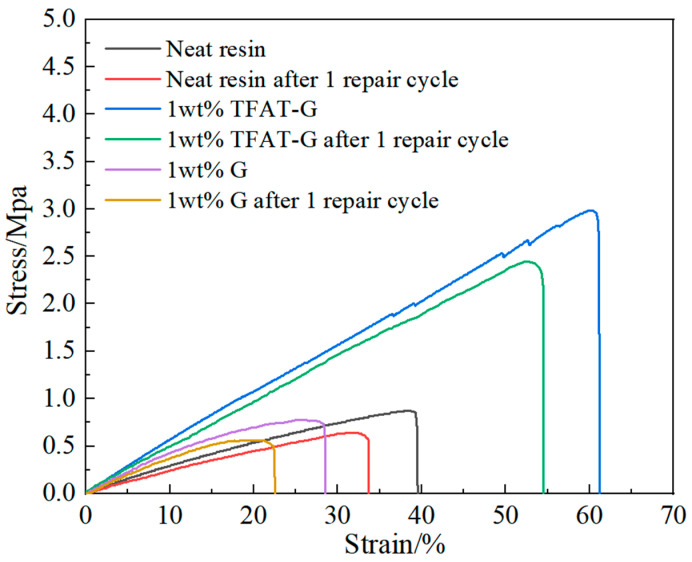
Stress-strain curve of resin nanocomposites before and after one repair cycle.

To compare the remaining stress strength, both the neat resin composites, 1 wt% G composites, and 1 wt% TFAT-G composites were tested using the method of GB/T 1050 before and after 1 repair cycle (Figure 13). All the composites were heated to 150 °C for 30 min as a repair cycle. After a repair cycle, the stress strength of 1 wt% TFAT-G composite is 280% higher than the neat resin, while the stress strength of 1 wt% G composite is only 72% of the neat resin. As a result, the addition of 1 wt% TFAT-G increased the stress strength of the neat resin composite and maintained 80% of its properties after one repair cycle.

**Figure 14:**
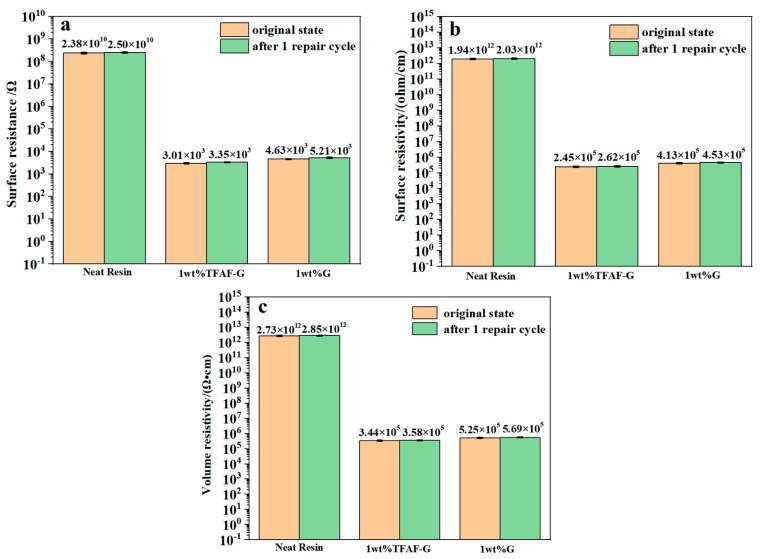
Resistivity of neat resin and nanocomposites before and after one repair cycle: (**a**) surface resistance, (**b**) surface resistivity, and (**c**) volume resistivity.

We tested the resistance with a resistivity tester, and the damaged part of the sample repaired by heating was measured. As the characterized conductivity of TFAT-G/resin composite was characterized, the remaining conductivity was tested after one repair cycle (Figure 14). The surface resistance, surface resistivity, and volume resistivity of the resin with 1 wt% TFAF-G were 3.01 × 10^3^ Ω, 2.45 × 10^5^ (ohm/cm), and 3.44 × 10^5^ (Ω·cm). After one repair cycle, the surface resistance, surface resistivity, and volume resistivity of the resin with 1 wt% TFAF-G were increased to 3.35× 10^3^ Ω, 2.62 × 10^5^ (ohm/cm), and 3.58 × 10^5^ (Ω·cm), respectively. The electrical conductivity of 1 wt% G nanocomposites was poorer than that of 1 wt% TFAT-G nanocomposites before and after repair. All of the neat resin composites, 1 wt% G composites, and 1 wt% TFAT-G composites demonstrated increased surface resistance, surface resistivity, and volume resistivity after one repair cycle.

## 4. Conclusions

In conclusion, we demonstrated nanocomposites with enhanced mechanical, self-repairing properties, and electrical conductivity based on Diels-Alder reversible crosslinking bonds. The covalently and reversibly crosslinked nanocomposites were prepared by Diels-Alder reactions using FA, DGEBA, BMI, and TFAT-G. Specifically, TFAT-modified graphene efficiently influenced enhancement, repeatability, and electrical conductivity. The conductivity of the composites became higher greater amounts of TFAT-G were added to the nanocomposites. The mechanical properties of the nanocomposites were enhanced the most when 1 wt% TFAT-G was added, with 233% increase in tensile strength, 63% increase in elongation at break, and 66% increase in Young’s modulus. Due to the poor dispersion of the unmodified graphene and resin matrix inside the composite, there is a lack of effective interface force between the two phases, which leads to the deterioration of the properties. Either the pure resin or the nanocomposites with 1 wt% TFAT-G added retained 83% of the original strength and 90% of the electrical conductivity after one heating repair cycle. We expect that such high-performance self-healing conductive composites will provide options for smart materials and potential applications in electronic engineering.

## Figures and Tables

**Table 1:** Mechanical properties of nanocomposites.

Sample	Tensile Strength/Mpa	Strain at Break/%	Modulus/Mpa
Neat resin	0.884 ± 0.103	38.348 ± 0.582	3.142 ± 0.177
0.25 wt% TFAT-G	1.389 ± 0.118	46.500 ± 2.354	4.238 ± 0.186
0.5 wt% TFAT-G	2.232 ± 0.175	51.333 ± 1.762	4.826 ± 0.332
0.75 wt% TFAT-G	2.581 ± 0.216	55.318 ± 2.461	5.031 ± 0.475
1 wt% TFAT-G	2.948 ± 0.181	62.333 ± 3.254	5.219 ± 0.494
1.25 wt% TFAT-G	2.437 ± 0.154	41.276 ± 2.292	5.743 ± 0.428
1 wt% G	0.779 ± 0.136	28.484 ± 2.953	4.338 ± 0.371
Neat resin after one repair cycle	0.643 ± 0.117	33.67 ± 1.286	2.749 ± 0.232
1 wt% TFAT-G after one repair cycle	2.447 ± 0.225	54.108 ± 2.318	5.014 ± 0.524
1 wt% G after one repair cycle	0.561 ± 0.125	22.451 ± 1.384	3.697 ± 0.531

## Data Availability

Data are contained within the article.
